# Inequalities in Environmental Cancer Risk and Carcinogen Exposures: A Scoping Review

**DOI:** 10.3390/ijerph20095718

**Published:** 2023-05-04

**Authors:** Kristian Larsen, Ela Rydz, Cheryl E. Peters

**Affiliations:** 1 Health Canada, Office of Environmental Health, Healthy Environments and Consumer Safety Branch, Environmental and Radiation Health Science Directorate, Ottawa, ON K1A 0K9, Canada; 2 CAREX Canada, School of Population and Public Health, University of British Columbia, Vancouver, BC V6T 1Z3, Canada; 3 Department of Geography and Planning, University of Toronto, Toronto, ON M5S 3G3, Canada; 4 Department of Geography and Environmental Studies, Toronto Metropolitan University, Toronto, ON M5B 2K3, Canada; 5 Cumming School of Medicine, University of Calgary, Calgary, AB T2N 4N1, Canada; 6 Prevention, Screening and Hereditary Cancer, BC Cancer, Vancouver, BC V5Z 4E6, Canada; 7 Population and Public Health, British Columbia Centre for Disease Control, Vancouver, BC V5Z 4R4, Canada

**Keywords:** inequalities, carcinogens, cancer risk, environmental exposures, built environment

## Abstract

**Background**: Cancer is the leading cause of death in Canada and a major cause of death worldwide. Environmental exposure to carcinogens and environments that may relate to health behaviors are important to examine as they can be modified to lower cancer risks. Built environments include aspects such as transit infrastructure, greenspace, food and tobacco environments, or land use, which may impact how people move, exercise, eat, and live. While environments may play a role in overall cancer risk, exposure to carcinogens or healthier environments is not equitably spread across space. Exposures to carcinogens commonly concentrate among socially and/or economically disadvantaged populations. While many studies have examined inequalities in exposure or cancer risk, this has commonly been for one exposure. **Methods**: This scoping review collected and synthesized research that examines inequities in carcinogenic environments and exposures. **Results**: This scoping review found that neighborhoods with higher proportions of low-income residents, racialized people, or same-sex couples had higher exposures to carcinogens and environments that may influence cancer risk. There are currently four main themes in research studying inequitable exposures: air pollution and hazardous substances, tobacco access, food access, and other aspects of the built environment, with most research still focusing on air pollution. **Conclusions**: More work is needed to understand how exposures to these four areas intersect with other factors to reduce inequities in exposures to support longer-term goals toward cancer prevention.

## 1. Introduction

Cancer is a major cause of death worldwide, which resulted in approximately 10 million deaths in 2020 [1]. In Canada, cancer is the leading cause of death, and cancer incidence and deaths are increasing as the population ages [2,3]. In 2022, an estimated 233,900 new cancer cases and 85,100 cancer deaths were expected in Canada [4]. In addition to the burden of illness, cancer care is costly, amounting to approximately 7.5 billion CAD in 2012 in Canada, over double the costs from 2005 [5]. There are also many indirect costs associated with cancer, including out-of-pocket costs, loss of earnings, and time costs. In total, the direct and indirect costs of cancer in 2021 in Canada were estimated at 26.2 billion CAD, with approximately one-third of this being borne by patients and their families [6]. These significant human health and economic impacts make cancer control a significant public health issue.

Environmental carcinogen exposure is an important cancer risk factor that can be modified. In Europe, an estimated 3.6% of lung cancers are due to air pollution exposure each year [7]; in Ontario, Canada, an estimated 5.8% of lung cancers are due to air pollution (PM_2.5_ and diesel particulate matter) [8]. When considering the most prevalent environmental carcinogen exposures in Ontario (solar ultraviolet radiation, radon, arsenic, acrylamide, asbestos, and select others), an estimated 3540–6510 cancers could have been prevented each year by controlling these exposures [8]. In addition, the built environment including aspects such as spatial proximity, transit infrastructure, greenspace, and land use can shape cancer risk by impacting how people move, exercise, eat, and live [9].

Importantly, exposure to environmental carcinogens is not evenly distributed across populations creating environmental inequity. Exposures are concentrated among socially and/or economically disadvantaged populations who may be especially vulnerable to hazardous exposures due to limited resources at the individual and community level [10]. For example, higher exposures to hazardous air pollutants have been observed in areas with greater concentrations of low-income and marginalized communities, including racialized people (Asian and Black) [11,12,13,14], same-sex male partner households [15], and isolated immigrants [16]. Disproportionate exposures among equity-deserving communities have also been observed for non-air-pollutant-related hazards, including water contaminants such as lead [17,18], lack of greenspace [19,20], and poor walkability scores [21,22].

Social demographics such as income, education, unemployment, housing conditions, and the neighborhood can play an integral role in several behavioral risk factors such as diet, physical activity and obesity, or substance use such as smoking and alcohol consumption [23,24,25,26], which are inherently related to certain types of cancer [27]. In the United States, over one-third of cancer deaths are attributed to diet, lack of physical activity, and obesity while another third relates to exposure to tobacco products [27]. Furthermore, in Europe, lower educational attainment is related to higher smoking rates, as well as lower physical activity levels and consumption of fruits and vegetables [28]. Sexual and gender minorities have been found to have higher smoking rates compared to their heterosexual counterparts in Canada and the United States [29], as well as increased risk factors for other types of cancers [30]. For example, an increased risk of anal cancer has been found among gay men, who are at increased risk of human papilloma virus infections of the anus, and an increased risk of breast and gynecological cancers has been noted among lesbian women, possibly due to fewer pregnancies and children, higher body mass indices, and less exercise, among other factors [31]. Social demographics may play a role in both higher carcinogen exposures and higher behavioral risk factors, increasing the odds of cancer for residents living in these environments.

The scope of environmental justice and environmental equality research is vast. Although previous reviews have investigated inequitable exposures via specific routes of exposure (e.g., indoor air pollution [32] and outdoor air pollution [33]), none, to our knowledge, have focused on carcinogenic exposures more broadly. CAREX (Carcinogen Exposure) Canada is a program of research that evaluates occupational and environmental carcinogen exposures in Canada by drawing on publicly available data sources [34]. Current estimates of environmental exposures include maps of predicted levels of specific carcinogens in Canada, as well as estimates of lifetime excess cancer risk [35]. These present a broad picture of Canadians’ general environmental exposures and do not capture the inequitable distribution of exposures across the social determinants of health. Thus, the objective of this scoping review was to collect and synthesize research that examines inequities in environmental exposures to carcinogens or environments that relate to cancer behavioral risk factors, relevant to the Canadian context. The overall goal of this inquiry is to support the development of new CAREX environmental exposure estimates that are policy-actionable from an equity, diversity, and inclusion perspective.

## 2. Materials and Methods

### Search and Selection Strategy

For this scoping review, we searched both PUBMED and SCOPUS for articles on 6 October 2021 with no restrictions for dates. The search terms were environment * AND (inequ * OR dispari *) AND (cancer OR carcinogen). All articles were imported into Covidence, which is online software that streamlines the review process. Inclusion criteria selected for articles written in English, that were not review articles, that examined carcinogen exposure/environment or cancer outcomes not including mortality, that mentioned inequalities or disparities, that were environmental in nature (i.e., not occupational), and that took place in Canada, the United States, New Zealand, Australia, or western Europe. While occupational exposures are undoubtedly an important route of exposure, they were beyond the scope of this review, as the focus was on exposures in the environment.

Two team members (E.R. and K.L.) independently reviewed the titles and abstracts to reduce bias in the selection of articles for inclusion, and any disagreements were resolved by the senior author (C.E.P.). This set of articles was then moved to full-text review in Covidence.

Reasons for excluding a paper during full-text review were the same as the initial inclusion criteria and were noted in Covidence, and any disagreements between the two main reviewers (E.R. and K.L.) were also resolved by a third reviewer (C.E.P.). Papers that made it through this stage were sent for data extraction in Covidence. Data extracted included study location, objective, design, population, and spatial scale. The methods used in the study were also examined with a focus on the data sources for both carcinogenic exposure and inequalities/disparities, as well as analytical methods. We also summarized the results, including sample size and observed cancer disparities/inequalities.

The extracted data were then summarized into more concise tables to identify themes and findings more easily. Summary values were calculated to describe the body of literature with regard to the exposures, inequalities, and outcomes examined.

## 3. Results

In total, 3016 papers were identified by the literature search strategy. After the removal of duplicates, a total of 2188 articles remained for title and abstract screening. Overall, the reviewers disagreed on the inclusion of 137 papers (agreement rate: 94%) and with the support of the third reviewer, consensus was reached on the included studies.

**Figure 1:**
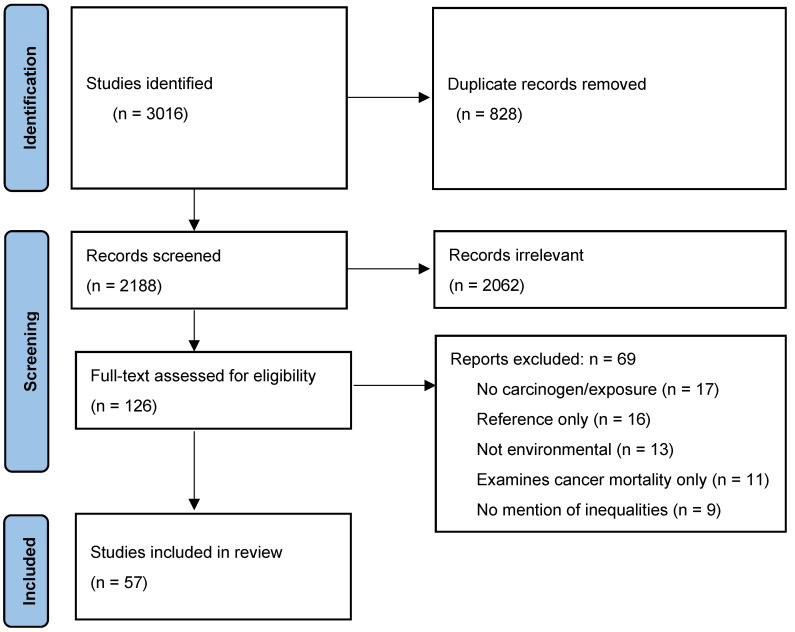
PRISMA diagram illustrating the article selection process.

After the title and abstract review, a total of 126 papers were included in full-text review during which they were assessed in more detail on the basis of the aforementioned inclusion criteria. A total of 69 papers were excluded (Figure 1). In total, 57 manuscripts were included in the data extraction process and analysis.

Overall, most of the studies were conducted in the United States (n = 46), with five from the United Kingdom, three from New Zealand, and one each from Australia, Canada, and France. These studies were conducted at varying spatial scales, with 26 local- or city-based studies, 19 national studies, and 12 regional studies (state, provincial, or multiple study sites). The majority of papers (n = 37) examined some aspect of air pollution (hazardous air pollution (HAP), particulate matter, diesel engine exhaust, or other air pollution measures). One study examined solar ultraviolet radiation (UVR), one examined nitrate in drinking water, and one study assessed perchloroethylene in buildings with dry-cleaning facilities. In total, 14 studies examined aspects of the built environment, with four studies examining more general aspects of the built environment, while an additional four assessed the food environment, four evaluated the tobacco environment, and two studied access to greenspace.

### Data Sources and Approach

To obtain demographic data to assess potential inequalities, the majority of papers relied on census data (n = 44), while eight studies used surveys, and the others used more local health-based studies, which may have included surveys and other measurement methods; one study assessed mortality records. Exposure data came from a variety of sources, with the US EPA’s NATA (National Air Toxics Assessment) being the most common (n = 29). Four studies used data on food outlet location/type (which was typically taken from business directories), four also assessed tobacco retailers’ data (obtained from government tobacco taxation records, national databases, or store types), three modeled air pollution datasets, two used land-use data, two used built environment data, and two used data from the EPA cumulative exposure dataset. The remaining studies used data from one of the following sources: environmental audit of playgrounds, California Air Resources Board health risk assessment, California Cancer Registry, California Neighborhoods Data System, community water system, measurement, National Pollutant Release Inventory (NPRI), Toxics Release Inventory (TRI), and National Ambient Air Quality Standards (NAAQS). The analytical approaches of the 57 studies were similar, with a modeling approach (e.g., GEE, OLS, linear, or logistic models) being applied in 48 of the studies. The remaining eight papers applied descriptive analyses, including *t*-tests, spatial clustering, and chi-squared analysis.

## 4. What Did the Studies Find?

### 4.1. Air Pollution and Hazardous Substances

The details for each manuscript were broken into two tables. Table 1 displays all papers related to hazardous air pollution, while Table 2 includes the other studies, which mainly focused on aspects of the built environment including greenspace, along with access to healthy/unhealthy food and tobacco. Results supported the hypothesis that inequalities in carcinogen exposures exist. The majority of studies examined inequalities with respect to socioeconomic status (SES) and/or race/ethnicity. As reported in Table 1, of the 41 studies that examined some form of air pollution or toxic substances, almost all of them found that race/ethnicity and/or SES was significantly related to cancer risk. While exposures and results did vary by race/ethnicity, many studies examined predominantly Black, Hispanic, or Asian neighborhoods, where exposure or lifetime excess cancer risk was frequently higher. Interestingly, two studies examined exposure for same-sex couples, and both reported that same-sex couples had higher exposures to environmental carcinogens or carcinogenic environments [15,36]. Padilla et al. (2004) was the only study that did not report consistent findings between exposure and equity-deserving populations but did report that environmental inequalities related to the urban development and immigration patterns within cities in France [37]. For the one study that examined perchloroethylene exposure, the most important factor related to exposure was having a dry cleaner within the building, and this was consistent regardless of socioeconomic status [38]. Lastly, drinking water had higher nitrate values in predominantly Hispanic neighborhoods versus non-Hispanic areas with community water systems [39].

### 4.2. Other Carcinogens or Environmental Cancer Risk Factors

Table 2 summarizes articles examining other known/potential carcinogens or environmental cancer behavioral risk factors including solar UVR exposure, food access, built environment, tobacco environment, and greenspace. For both tobacco and food, access was typically defined by proximity, either by distance to outlets or density, which assesses clustering or the number of outlets. Access to quality foods typically examined how easily residents have access to healthy food (i.e., supermarket or fruit/vegetable stand) and less healthy food such as fast-food restaurants. Greenspace was typically examined with a proximity and density calculation (i.e., distance to or density of parks/open space) but was also sometimes assessed using a vegetation index. Other built environment characteristics examined neighborhood walkability, street connectivity, traffic, sidewalks, or other aspects that may encourage or discourage physical activity. Only one study examined solar UVR exposure via access to shade structures in parks and reported that lower-SES areas had poorer access to shade than their wealthier counterparts [84]. Greater spatial exposure to fast-food restaurants was associated with higher fast-food consumption and odds of obesity [80], especially for those in the lowest income category [80]. Lower-income areas commonly had more exposure to fast-food outlets [81,82]. More general built environment measures (such as walkability and greenspace) reported varying results related to exposures and disparities. For example, one study reported breast cancer risk to be highest for high income neighborhoods, with White women having the highest odds [75], and with adjustments for more urban environment and mixed land uses decreasing the disparities for all Black and Hispanic, but not White neighborhoods. One study reported that SES, along with race/ethnicity, were not related to physical activity levels for youth [78] while, another found that socioeconomic status and some aspects of the built environment were related to obesity [83]. For tobacco environments, the highest exposure tended to occur in predominantly Black and Hispanic neighborhoods [85,87,88] or areas of lower income [86]. Access to greenspace was typically lower for those in lower-SES neighborhoods [74].

## 5. Discussion

This scoping review found that neighborhoods with higher proportions of low-income residents, racialized people (e.g., Black, Hispanic, Asian), or same-sex couples had higher exposures to carcinogens and environments that may influence cancer risk. This review summarizes the available literature to examine carcinogenic exposure overall, including greenspace, food or tobacco access, solar UVR exposure, perchloroethylene, and other aspects of the built environment. The four general themes related to inequitable carcinogen exposures or environments that may relate to behavioral risk factors for cancer were air pollution and hazardous substances, access to healthy/unhealthy food, access to tobacco outlets, and more general built environment factors (i.e., walkability and access to parks/greenspace). However, the majority of studies assessing inequitable environmental exposures focused on air pollution, with little attention paid to other carcinogenic exposures or environments.

### 5.1. Air Pollution

Among studies of air pollution and exposure to hazardous substances, lower-income neighborhoods and/or those with a higher proportion of Black and Hispanic people commonly had higher exposures. While Black and Hispanic populations commonly had higher exposure in the United States [43,47,57,64], other countries reported similar findings for racialized populations relevant to their country [14,41]. In New Zealand, for instance, Asian and Māori populations had higher exposure to air pollution than their White counterparts [41]. One exception was reported in France, where inconsistent results were found across the four cities examined (Lille, Lyon, Marseille, and Paris), with some cities reporting evidence of inequities in racialized populations while others did not [37]. This demonstrates how the racial/ethnic and SES makeup of cities along with historical socioeconomics, immigration, and development patterns may impact exposures. For example, in Paris, census blocks with the highest income (or social status) had the highest exposure to nitrogen dioxide [37]. This finding is similar to results reported in Rome, Italy [89]. The authors suggested that these changes in exposures for higher-income areas reflect how development patterns have changed [37]. Air pollution, in some cases, is no longer largely originating from industrial emissions but rather from tail-pipe emissions from traffic, commuting, and movement of goods [37]. In France, larger industrial polluters have moved outside of Paris to other regions of France or even into Eastern Europe or developing countries [37]. Thus, air pollution in central Paris is mainly from transportation sources. On the other hand, in both Lille and Marseille, higher NO_2_ levels were reported in areas of highest distress (lowest income). Both Lille and Marseille were historically cities with large industrial economies. Currently, Lille has a textile industry, while Marseille has a large port along with steel and petrochemical industries [37]. The more industrial nature of these cities may relate to the higher exposures experienced closer to city cores, which is different from the situation in Paris.

In Paris and Lyon, exposure was highest in the higher-income areas, related to individual choices in which people prefer to live in particular neighborhoods within the city, where the benefits of that location (such as walkability, access to healthy foods or greenspace, etc.) may outweigh the detriment of higher air pollution exposure [37]. For example, many urban areas may have higher traffic-related air pollution, but may also have better access to healthy foods, fewer tobacco outlets, and more walkable neighborhoods with greenspace and parks. The intersectional nature of human and urban development patterns, environmental contamination, and categories of marginalization are important considerations that may vary substantially by region or jurisdiction.

### 5.2. Greenspace

Two studies examined access to greenspace, an aspect of the built environment, and both found that lower SES areas had poorer access to greenspace [73,74]. This is a common finding across the environmental health literature, with many studies linking lower-income areas with poorer access to parks [90,91,92]. This suggests that cities are being built or perhaps modified in an inequitable manner, allowing for parks and better greenspace in higher-income areas. It is unclear whether parks or greenspaces are being constructed with higher-income communities or being added after the fact, but overall access to greenspace and parks was inequitably distributed. With regard to health outcomes, one study from a review based in New Zealand did not find a relationship between greenspace and any health outcome (cancer or cardiovascular disease) [74], but the United Kingdom study did find a relationship with all-cause mortality [73]. Even though direct links between greenspace and cancer risk were not reported, this is an active area of investigation, and a tentative association has been noted in the broader literature. Greenspace is thought to have several health-promoting benefits [93]. This may relate to escapes from noise and pollution [94], reductions in the urban heat island effect [95], helping with anxiety levels [96] and/or overall mental health [97], improved air quality, and opportunities for physical activity [98]. Overall, the included studies demonstrate that more general interventions at the population level (i.e., changing access to or improving greenspace) will more effectively impact health behaviors than individual-level interventions [73,99]. Thus, a continued examination of the association between greenspace and cancer, and the inequitable access to greenspace will be important moving forward from a cancer prevention and health inequities perspective.

### 5.3. Access to Food

Inequities, especially related to income, in access to healthy food have been discussed since the early 2000s [100,101,102,103]. The clustering of fast-food outlets (also referred to as food swamps) has also been reported more frequently in equity-deserving neighborhoods [104,105]. Food access can impact cancer risk since diets low in fruits and vegetables and higher in processed foods are known risk factors for several types of cancer; it is also related to obesity and overweight, which are inherent risk factors for several cancers. Studies reviewed in this paper found that inequalities in access to food exist [79,80,81,82], as lower-SES areas commonly had the highest exposure to fast-food restaurants. Furthermore, odds of obesity were also higher for those with low income and the highest fast-food exposure [79,80]. However, evidence in the broader literature regarding the relationship between food environment and dietary consumption is inconclusive at this point, partly due to varying methodologies and issues defining “access” [106], along with cultural and policy-based differences in the various countries examined. Even so, several studies included in this review reported positive associations between fast-food exposure and both fast-food consumption [107,108,109] and body weight [110,111,112]. Thus, when considering environmental cancer risk and potential inequities, healthy food access may be an important piece to consider.

### 5.4. Access to Tobacco

Similar to the effects of food environments, neighborhoods with a higher density of tobacco outlets may lead to a higher purchasing of cigarettes and other tobacco-related products [113,114] and underage sales [115]. The density of tobacco outlets has also been linked to higher tobacco usage among adolescents, with lower-income neighborhoods having higher odds of smoking [116]. As reported in Table 2, race/ethnicity also played a role in tobacco access, meaning those living in predominantly Black or Hispanic areas were also overexposed, in addition to low-income residents. Tobacco remains the highest modifiable contributor to the risk for many cancers, and public health campaigns over the past several decades have been effective at lowering rates of smoking. Findings from this review suggest that smoking prevention policies should better investigate ways to more effectively reach and support people of lower SES in tobacco use reduction or elimination. It has been reported that longer distances (i.e., lower proximity) to tobacco outlets was an effective method to reduce smoking [117], but the density of outlets or clustering was not a significant factor. This suggests that not having nearby access to tobacco may be beneficial to lower smoking rates, but the feasibility of this as a policy idea is not established.

### 5.5. Complexities, Challenges, and Future Work

One important aspect to discuss, which is particularly pertinent for studies that examine the built environment, is that the data sources, populations under investigation, and methods are commonly different. Many studies examining access to food, walkability, physical activity, or obesity have produced mixed results, with some studies reporting positive associations, some others reporting negative associations, and some detecting significant relationships [74,75,78,80,82,83,85,86,87,118]. Given the scope of these studies, with many examining different populations, in various countries, the results are not always generalizable to each country, neighborhood, or even city. Furthermore, the methods and data utilized to examine potential relationships are also inconsistent, further complicating interpretation.

One apparently contradictory finding from our review is that White women had higher odds of breast cancer, especially in high-income neighborhoods [75]. Breast cancer epidemiology is complex, and, while there are links reported between environmental chemical exposures and the risk of breast cancer, there are several social factors relating to risk that tend to cluster in wealthier women [119,120]. These include a higher likelihood of remaining childfree, delaying childbirth until older age, use of hormone supplements, and less frequent breastfeeding [121]. Furthermore, certain genetic predispositions may put certain White populations at a higher risk of breast cancer [121]. While higher-income White women may have higher incidence rates for breast cancer, mortality rates are commonly higher among Black women [122], which likely relates to access to primary care and specialist physicians, cancer screening programs, or the type of breast cancer itself [123,124].

An important observation to make is that while we were able to discern four main themes in this scoping review (air pollution, access to foods, access to tobacco, and built environment), it is highly likely that these intersect in complex ways with environmental carcinogen exposures, human behavior, and cultural dynamics, but the topics were typically considered in isolation (i.e., independent of one another). Future work should take a broader approach to examine carcinogen exposures and the complex intersections of environmental contamination, the work people do, wealth and income inequality, racism, and cultural sensitivity. It is important to contextualize carcinogenic exposures in order to gain a better understanding of potential cancer and/or other health risks associated with the environment. This review adds to the literature by looking beyond the individual exposures, but more empirical research is needed to further fill these gaps.

Furthermore, complexities exist in how we define “healthy” environments, as neighborhoods can have, for example, higher exposure to fast food but also good access to parks, greenspace, and walkable streets. Others may have higher air pollution, while other environmental aspects of neighborhoods, such as parks, high-quality food access, or reduced tobacco access, may be protective. This may become a more common occurrence in many cities as pollution becomes more related to transportation (i.e., cars and trucks) than industrial emissions, and more walkable, older neighborhoods may experience more traffic-related air pollution.

One of the limitations of this review was the fact that most studies were completed in the United States and, thus, may not always be generalizable to Canada. While this is a limitation, it also highlights the need for future work to examine inequities in exposures in other countries to gain a better understanding of how exposure varies by country, region, city, or neighborhood. Furthermore, most of the reviewed studies were cross-sectional and ecological in nature with analysis conducted at the census tract level (or similar census boundary).

Findings from this scoping review highlighted that many lower-SES areas or neighborhoods with a higher proportion of racialized people commonly have higher exposures to carcinogens or environments that may influence behavioral risk factors for cancer. In an effort to examine these issues at the national level, as part of the CAREX Canada mandate, our next steps are to update our environmental estimates and knowledge products to summarize neighborhood-level exposures with an equity lens. Our renewed purpose as a result of this work is to generate data-driven knowledge products that can be used by policymakers and advocates to reduce inequities in spatially clustered cancer risk factors in Canada.

## 6. Conclusions

This scoping review synthesized research examining inequities in environmental exposures to carcinogens. The current literature examining inequitable carcinogenic exposures can be summarized into four main themes: air pollution and hazardous substances, tobacco access, food access, and other aspects of the built environment, although studies examining hazardous air pollutants are by far the most common. Findings from this review highlighted that, while some differences exist, neighborhoods with a higher percentage of lower-income and/or racialized residents typically have higher carcinogen exposures, as well as lower exposure to healthy built environments. Inequities in environmental cancer risk need to be examined and addressed by policymakers to address systemic factors influencing environmental risks related to cancer and other chronic diseases. Steps taken to improve the environment now will support longer-term goals toward cancer prevention.

## Figures and Tables

**Table 1:** Summary of studies examining air pollution and hazardous substances.

Author, Year, Location	Study Objective	Exposure	Study Population and Data Source	Disparities	Results Summary *
Chakraborty 2014, Houston, USA [40]	To determine if chronic and acute pollution risks in the Greater Houston area are distributed inequitably, and if inequities differ by source of exposure	Acute/chronic pollutant Chemical releases Lifetime excess cancer risk	Census data from residents of the Greater Houston area, must have at least 500 persons and 50 housing units	Race/ethnicity, SES/income, language	Neighborhoods with a higher percentage of Hispanic residents, lower percentage of homeowners, and higher income inequality have greater exposure to both chronic and acute pollution risks.
Pearce 2006, Christchurch, New Zealand [41]	To assess if there is a social/ethnic gradient in exposure to air pollution from domestic heating	Air pollution from domestic heating dispersion model	Census data for residents of Christchurch	Race/ethnicity, SES/income	Higher pollution levels for Asian and Māori populations and economically disadvantaged communities.
Yu 2016, Tampa Bay, USA [42]	To estimate emission concentrations and exposures to improve understanding of impacts of urban design on exposure disparities	Air pollution outdoor air (benzene, 1,3-butadiene, formaldehyde, acetaldehyde, and nitrogen oxide)	Census data for residents of Hillsborough county	Race/ethnicity SES/income	Black, Hispanic, and lower-income residents had higher exposure to benzene, 1,3-butadiene, and nitrogen oxide, but lower exposure to acetaldehyde and formaldehyde.
Rosenbaum 2011, USA [43]	To compare diesel inhalation intake across harbor areas in the US, and to estimate the size and demographic composition of populations who are at increased carcinogenic risk	Diesel engine exhaust near 43 US marine rail harbors	Census data for people living in 43 US marine harbor areas where carcinogenic health risk exceeds 10 per million	Race/ethnicity, SES/income	Low-income households and Hispanic or Black residents have higher exposure to diesel engine exhaust.
Hricko 2014, California, USA [44]	To describe cancer risks for residents living close to major rail yards with emissions of diesel, and to identify potential racial and income disparities in exposure	Diesel engine exhaust near rail yards Lifetime cancer risk	Census data for residents with high diesel cancer risks (100+ in a million) and living near railyards in California	Race/ethnicity, SES/income	Overall higher risk of living near railyards and high diesel cancer risks for Black and Hispanic residents and lower-income groups.
Osiecki 2013, Cook County Illinois, USA [45]	To examine spatial associations and geographic patterns of sociodemographic characteristics, environmental cancer risk, and cancer rate	Exposure to hazardous sites Cancer risk	Census data for west and south regions of Chicago	Race/ethnicity, SES/income, poverty, home ownership, education	Areas with high poverty and high proportions of Black residents had higher environmental cancer risk.
James 2012, Cancer Alley Louisiana, USA [46]	To examine race- and income-based disparities in cancer risks from air toxics in Cancer Alley, LA	Hazardous air pollutants (formaldehyde, benzene, acetaldehyde, carbon tetrachloride, ethylene oxide, 1,3-butadiene, and naphthalene) Lifetime cancer risk	Census data for those living in cancer alley	Race/ethnicity, SES/income, education, lone parenthood	Higher lifetime cancer risks for Black and lower-income residents. Formaldehyde and benzene were the two largest contributors to the disparities.
Stoner 2013, USA [47]	To evaluate whether exposure to outdoor air toxics in early childhood increased asthma risk or severity	Hazardous air pollutants (modeled) Asthma prevalence	Early child longitudinal study, born in 2001, with mothers ≤15 years old	Race/ethnicity, SES/income	Higher exposure to air toxins for Black, Hispanic, and low-income residents.
Wilson 2015, South Carolina, USA [48]	To study cancer risk disparities from exposure to hazardous air pollutants in South Carolina	Hazardous air pollutants (modeled) Cancer risk	Census data for residents of South Carolina	Race/ethnicity, SES/income, home ownership	Cancer risk was higher in census tracts with higher percentage unemployed, percentage in poverty, lower per capita income, and higher percentage of non-White residents; negative associations for homeownership and education.
Lievanos 2019, USA [49]	To identify to what extent hazardous air concentrations impact marginalized Indigenous peoples, Whites, Blacks, and Latinxs, as well as to what extent APIs affect the probability of exposure to carcinogenic air pollution clusters	Hazardous air pollutants (modeled) Lifetime cancer risk	Census data	Race/ethnicity	Indigenous residents had higher exposures in mid-Atlantic region; overall, Black, Asian/Pacific Island, or Hispanic residents had higher exposures to carcinogenic air pollution clusters.
Collins 2017, USA [15]	To examine disparities in exposure to hazardous air pollutants and risk of cancer or respiratory health among same-sex partners	Hazardous air pollutants (modeled) Lifetime cancer risk	Census data and American Community Survey	Race/ethnicity, SES/income	Same-sex partners had higher lifetime cancer risk.
Rubio 2020, USA [50]	To study ancestry-based ethnic inequalities among Americans at the national level	Hazardous air pollutants (modeled) Lifetime cancer risk	Census data for Americans from 26 ancestries	Race/ethnicity, immigration	Americans of Dominican, Ethiopian, and Somalian descent had the highest total lifetime cancer risks.
Pastor Jr 2005, California, USA [51]	To identify environmental inequalities in exposure by race and income to hazardous air pollutants in California	Hazardous air pollutants (modeled) Lifetime cancer risk	Census data for California residents	Land uses/income, race/ethnicity	Higher lifetime cancer risks for Black, Hispanic, and Asian/Pacific Island residents.
Liecvanos 2015, USA [16]	To assess where air toxic health risk clusters are in the US, and to study the relationship between air-toxic health risk clusters and race, class, and immigrant status	Hazardous air pollutants (modeled) Lifetime cancer risk	Census data for continental USA	Race/ethnicity, SES/income, immigration	Black, Hispanic, and Asian/Pacific Island residents and lower-income residents were at higher risk for living in local air toxic clusters and higher lifetime cancer risk.
Grineski 2019, USA [52]	To identify geographical hotspots of lifetime cancer risk from hazardous air pollutants, as well as social disparities in the US by school district	Hazardous air pollutants (modeled) Lifetime cancer risk	Census data for people ≤ 18 years old	Race/ethnicity, SES/income, immigration	Considering all exposure sources, lifetime cancer risk increases with higher proportion of children in poverty, with disability, and that are foreign-born, Black, and multiracial/other, but decreases with increased proportion of American Indian children and decreased proportion of American Indians.
Grineski 2019, Honolulu, Los Angeles, San Francisco, Seattle, USA [53]	To study disparities in residential exposure to carcinogenic hazardous air pollutants among Asian Americans	Hazardous air pollutants (modeled) Lifetime cancer risk	Census data for residents of Honolulu, Los Angeles, San Francisco and Seattle area	Race/ethnicity, SES/income	Korean ancestry was positively associated with lifetime cancer risk in Los Angeles. Chinese ancestry was positively associated in Los Angeles and Honolulu, but negative in Seattle. Japanese ancestry was positively associated with lifetime cancer risk in San Francisco and Seattle. South Asian ancestry was negatively associated with lifetime cancer risk in Seattle and Honolulu. Filipino ancestry was positively negatively in Honolulu, Los Angeles, and Seattle, but negative in San Francisco.
Linder 2008, Houston and Harris County Texas, USA [54]	To examine the spatial distribution of cumulative, air-pollution-related cancer risks, and to identify ethnic, economic, and social disparities	Hazardous air pollutants (modeled) Lifetime cancer risk	Census data for residents of Houston and Harris county	Race/ethnicity, SES/income, unemployment, education	Higher lifetime cancer risk for areas with higher proportions of Hispanic residents, and those living in poverty, with less than high-school education.
Jia 2014, Memphis Shelby County Tennessee, USA [55]	To assess the relationship between racial composition and cancer risks from air toxics exposure	Hazardous air pollutants (modeled) Lifetime cancer risk	Census data for residents of Memphis and Shelby County	Race/ethnicity, SES/income	Higher lifetime cancer risk for census tracts with higher proportion of Black residents. The distribution of major roads and industrial facilities caused the largest disparities.
Morello-Frosch 2001, Southern California, USA [56]	To assess lifetime cancer risks associated with hazardous air pollutants, and to determine if there are racial and economic differences in cancer risk	Hazardous air pollutants (modeled) Lifetime cancer risk	Census data for residents of southern California	Race/ethnicity, SES/Income	Differential lifetime cancer risks observed by race, with Black, Hispanic, and Asian residents having the highest risk.
Morello-Frosch 2006, USA [57]	To assess if racial and economic disparities in estimated cancer risk associated with air toxics are modified by levels of residential segregation	Hazardous air pollutants (modeled) Lifetime cancer risk	Census data for residents of US metropolitan areas	Race/ethnicity, poverty, material deprivation	Differential lifetime cancer risks observed by race, with Hispanic residents having the strongest relationship.
Collins 2011, El Paso County Texas, USA [58]	To assess contextually relevant variables, and intra-racial/ethnic variables in the study of inequitable distribution of air toxic exposures	Hazardous air pollutants (modeled) Lifetime cancer risk	Census data from El Paso county	Race/ethnicity, language, citizenship	Higher lifetime cancer risk for block groups with increased proportion of residents who are Hispanic, without high-school diploma, income below poverty line, renter-occupied, female-headed households, poor English proficiency, and foreign-born, and in block groups with the lowest median household income.
Collins 2015, Greater Houston, USA [59]	To assess if cancer risks from outdoor hazardous air pollutant exposures are distributed inequitably and if having a disadvantaged racial minority study modifies the effects on cancer risk	Hazardous air pollutants (modeled) Lifetime cancer risk	Census data from residents of the Greater Houston area	Race/ethnicity, SES/income, housing location	Black and Hispanic residents had higher lifetime cancer risks.
Collins 2017, Greater Houston, USA [15]	To test for disparities in hazardous air pollutants on the basis of census tract composition of same-sex partner households	Hazardous air pollutants (modeled) Lifetime cancer risk	Census data from residents of the Greater Houston area, at least 500 people or 200 households	Race/ethnicity, SES/income, home ownership	Same-sex partners had higher lifetime cancer risk.
Ekenga 2019, St. Louis Metropolitan Area, USA [60]	To study the relationship between residential segregation and neighborhood sociodemographic characteristics and cancer risk from air toxins	Hazardous air pollutants (modeled) Lifetime cancer risk	Census data from residents of the Greater St. Louis area	Race/ethnicity, SES/income	Exposure to carcinogenic air pollution higher for neighborhoods with higher proportion of residents who are Black, in poverty, or unemployed, and who have low education.
Loustaunau 2019, Harris County Texas, USA [61]	To assess how cancer risk form exposure to on-road hazardous air pollutants differs across and within each major racial/ethnic group	Hazardous air pollutants (modeled) Lifetime cancer risk	Census data restricted to census tracts of ≤500 people	Race/ethnicity, SES/income, poverty, homeownership, education, language	Higher lifetime cancer risk for Black and Hispanic residents.
Grineski 2017, USA [13]	To study disparities in residential hazardous air pollutant exposures among Asian Americans	Hazardous air pollutants (modeled) Lifetime cancer risk	Census tracts with at least 500 people, 200 households	Race/ethnicity, SES/income, home ownership	Higher lifetime cancer risk for Chinese, Korean, and South Asian residents.
Morello-Frosch 2002, Los Angeles, USA [62]	To identify disparities in ambient air toxics exposures among school children in the LA Unified School District	Hazardous air pollutants (modeled) Lifetime cancer risk among school children	California basic education survey data for school children in Los Angeles unified school district	Race/ethnicity, SES/income	Higher lifetime cancer risks for Black and Hispanic residents.
Apelberg 2005, Maryland, USA [63]	To evaluate disparities in estimated cancer risk from exposure to air toxics by emission source category	Hazardous air pollutants (modeled) Lifetime excess cancer risk	All individuals in Maryland in the census	Race/ethnicity, SES/income, education	Income related to lifetime cancer risk up until 50,000 USD; predominantly Black neighborhoods had a higher lifetime cancer risk, but no relationship was observed for Hispanic neighborhoods.
Alvarez 2021, USA [64]	To estimate the intersectional effects of environmental health hazards at a structural or neighborhood level	Hazardous air pollutants (modeled) Lifetime excess cancer risk	Census data	Race/ethnicity, SES/income, female households, education	Higher lifetime cancer risk for Black and Hispanic residents, as well as low-income, low-education, and female-headed households.
Chakraborty 2009, Tampa Bay, USA [65]	To evaluate socio-spatial inequities in the distribution of cancer and noncancer risks associated with outdoor exposure to hazardous air pollutants emitted by on-road vehicles	Hazardous air pollutants (modeled) Lifetime excess cancer risk	Census data from residents of the Tampa Bay Metropolitan Statistical Area	Race/ethnicity, SES/income, transportation disadvantage	Higher lifetime cancer risks for Black and Hispanic residents, poverty, and no vehicle ownership.
Larsen 2020, North Carolina, USA [66]	To better understand how joint exposure to environmental and economic factors influence cancer	Hazardous air pollutants (modeled) Modelled cancer risk	Census data for residents of North Carolina	Race/ethnicity, SES/income	Higher pollution and lifetime cancer risk for SES disadvantage and higher Black population density.
Chakraborty 2017, Miami, USA [67]	To assess whether excess cancer risks due to ambient exposure to on-road mobile sources are distributed inequitably	Hazardous air pollutants (modeled) from vehicles Lifetime cancer risk	Census and survey data from adult residents in Miami area	Race/ethnicity, SES/income, rent status, language, immigration, unemployment	Higher lifetime cancer risks for Black and Hispanic residents, lower SES, and renters.
Collins 2015, Miami, USA [68]	To assess if cancer risks from on-road hazardous air pollutant exposures are distributed inequitably by household-level factors, and if inequities differ	Hazardous air pollutants (modeled) from vehicles Lifetime excess cancer risk	Census and survey data from adult residents in Miami area	Race/ethnicity, SES/income, housing location	Higher lifetime cancer risks for residents who are Black and Hispanic, lower-income, unemployed, and renting.
Chakraborty 2012, Tampa Bay, USA [69]	To evaluate spatial and social inequities in potential cancer risk from inhalation exposure to hazardous air pollutants from four types of emission sources	Hazardous air pollutants (modeled) from vehicles Lifetime excess cancer risk	Census data from residents of the Tampa Bay area	Race/ethnicity, SES/income, old age	Proportion of Black and Hispanic population was significantly associated with lifetime cancer risk, while proportion of owner-occupied homes was negatively associated.
Kershaw 2013, Toronto, Canada [14]	To inform priority-setting for pollution prevention by characterizing neighborhoods near large industrial air polluters	Hazardous air pollution near large emitters (kernel density estimates)	Census data for residents of Toronto	Race/ethnicity, SES/income, home ownership, unemployment, proportion children/seniors	Distance to pollution was significantly shorter for low-income individuals.
Huang 2017, USA [70]	To demonstrate the utility of unsupervised machine learning technique in identifying multiple chemical and non-chemical exposures	Hazardous exposures (acetaldehyde, benzene, cyanide, diesel PM, toluene, and 1,3-butadiene)	Census data	Race/ethnicity, SES/income, single mothers, education, sex	Census tracts with a high percentage of racial/ethnic people and low-income residents had higher estimated chemical exposure concentrations (fourth quartile) for diesel PM, 1,3-butadiene, and toluene.
Pastor Jr 2002, Los Angeles, USA [71]	To evaluate the demographic distribution of potentially hazardous facilities and health risks associated with ambient air toxics exposures among public school children	Hazardous sites Lifetime cancer risks for school children	California basic education survey data for school children in Los Angeles school district	Race/ethnicity, SES/income	School districts are more likely to be in census tracts with hazardous facilities, but have lower cancer risks. Hispanic students are more likely to attend schools near hazardous facilities, and have high cancer risk.
Padilla 2014, Lille, Lyon, Marseille and Paris, France [37]	To identify whether urban neighborhoods have uneven distribution of ambient air concentrations of nitrogen dioxide and deprivation in four French metropolitan areas	Nitrogen dioxide air pollution	Census data for residents of Lille, Lyon, Marseille, and Paris	Immigration, SES/income, job type, education	No consistent findings between exposure and deprivation.
Su 2009, Los Angeles, USA [72]	To propose a method for creating an index capable of summarizing racial/ethnic and socioeconomic inequalities from the impact of cumulative environmental hazards	Nitrogen dioxide and particulate matter air pollution Environmental hazard index	Census data for residents of Los Angeles	Race/ethnicity, poverty	Modest inequalities exist for environmental hazards in Los Angeles. The highest exposures were observed for non-White and low-SES residents.
Schaider 2019, USA [39]	To identify determinants of nitrate concentrations in US community water systems and to evaluate disparities	Nitrate concentrations in community drinking water systems	Americans served by the community water systems, population level for 39,466 counties	Race/ethnicity, poverty, home ownership	Higher nitrate values in predominantly Hispanic neighborhoods.
Storm 2013, New York City, USA [38]	To assess perchloroethylene exposures associated with dry cleaners in residential buildings, and to evaluate whether a disparity is present	Perchloroethylene exposures via dry-cleaning business presence in residential buildings	Residents from buildings with or without dry cleaner; at least one eligible adult (2–64 years) and child (5–14 years)	Race/ethnicity, SES/income	In buildings with dry cleaners, indoor air levels were five high times higher in predominantly Black and/or Hispanic neighborhoods and six times higher in low-income neighborhoods.

* All variables were statistically significant.

**Table 2:** Summary of papers studying remaining exposures.

Author, Year	Study Objective	Exposure	Study Population and Data Source	Disparities	Results Summary
Mitchell 2008, England, UK [73]	To examine income-related health inequality in populations living areas with differing amounts of greenspace	Access to greenspace	UK mortality records, those older than retirement age were excluded	SES/income	Mortality rates are higher in lower-SES areas with low access to green space.
Richardson 2010, New Zealand [74]	To examine the mechanisms via which greenspace availability may influence mortality outcomes, by contrasting health associations for different types of green space	Access to greenspace	Individual-level mortality data for every death between 1996 and 2005 from NZ Ministry of Health and restricted to urban areas	SES/income	Low-SES areas had lower access to total green space; outcome did not relate to cancer or caridovascular disease.
Conroy 2017, California, USA [75]	To examine breast cancer risk in African American and foreign-born Hispanics and the extent to which social and built environment characteristics explained the SES associations	Built environment (population density, crowded households, commute by car, number of parks, number of recreational facilities, street connectivity, fast-food vs. all restaurants, number of convenience stores, liquor stores, and fast-food restaurants vs. supermarkets and farmers markets) Breast cancer risk	Pooled data from the San Francisco Bay Area Breast Cancer Study and Cancer Registries	Income	High-income neighborhoods had higher risk of breast cancer. White women had the highest odds, followed by Hispanic and Black. Adjustment for urban and mixed-land use characteristics decreased the SES differences.
DeRouen 2018, San Francisco Bay Area, USA [76]	To assess if individual-level factors interact with neighborhood-level social and built environment factors to influence prostate cancer risk	Built environment (population density, mode of travel to work, residential mobility, household crowding, street connectivity, businesses, fast-food restaurants vs. all restaurants, convenience stores, liquor stores, and fast-food restaurants vs. supermarkets and farmers markets, parks, farmers markets, traffic density)	African American and white men from the San Francisco Bay Area, aged 40–79	SES/income	Higher-SES neighborhoods had an increased risk of prostate cancer. Higher education was protective against advanced disease in low-SES neighborhoods, but had no impact in higher-SES neighborhoods. For localized disease, the SES was largely explained by known prostate cancer risk factors and environmental factors, as well as population density, crowding, and residential mobility.
Gomez 2011, California, USA [77]	To develop the California Neighborhoods Data System to examine neighborhood characteristics on cancer incidence and outcomes in populations	Built environment for cancer risk	Population level with use of census data	Race/ethnicity, SES/income	SES was related to cancer rates, as well as residential crowding, percentage foreign-born, English knowledge, education, poverty, housing value, and gross rent. Ethnicity was related to cancer rates, SES, and exposures.
Shams-White 2021, USA [78]	The purpose of this study was to examine associations of home neighborhood environmental factors with moderate to vigorous physical activity (MVPA) among a national sample of adolescents	Built environment for youth physical activity	Survey of dyads for parents and adolescents (aged 12–17); parents lived with adolescent at least 50% of time	Race/ethnicity, education	SES and race/ethnicity were not significant for MVPA. Living in higher-density neighborhoods and neighborhoods with older homes was positively associated with adolescent MVPA. Living in neighborhoods with shorter commute times was negatively associated with MVPA.
Burgoine 2016, Greater London, UK [79]	To assess if education modifies associations beween fast-food consumption and body weight, with respect to home and work neighborhood fast-food outlet exposure	Density of fast-food restaurants within 1 mile of home and work Access to supermarkets	Participants born between 1950 and 1975 completed surveys for Fenland cohort study	Educational attainment	Greater fast-food consumption, BMI, and odds of obesity were associated with greater fast-food outlet exposure and a lower educational level. High fast-food outlet exposure amplified differences across levels of education.
Burgoine 2018, Cambridgeshire County, UK [80]	To examine associations of neighborhood fast-food outlet exposure and household income on frequency of consumption of processed meat	Fast-food environment (proportion of fast-food outlets vs. all food outlets) Food consumption	Adults aged 38–72 registered with NHS lives within 25 miles of UK assessment centres in London	SES/income	Income and fast-food proportion were associated with BMI, body fat, obesity, and frequent processed meat consumption. Odds of obesity were greater for lowest-income participants and for those most exposed to fast-food outlets
Maguire 2015, Norfolk, UK [81]	To assess the area-level density of takeaway food outlets and presence of supermarkets with respect to deprivation over time and to examine deprivation-specific food environment stability	Fast-food environment Supermarket access	Examined store locations and types over time (1990–2008)	SES/income	Lowest-SES arreas had highest density of fast-food outlets.
Maguire 2017, Fenland and East Cambridgeshire, UK [82]	To compare socioeconomic differences in foodscape exposure using a number of commonly used GIS-based metrics to better understand the implications of selecting different metrics	Fast-food environment Supermarket access	Population based cohort aged 30–62 at recruitment from Fenland Study	SES/income, education	Lower-SES areas had highest percentage of fast-food outlets.
Conroy 2018, Hawaii and California, USA [83]	To examine the associations of obesity with attributes of the social and built environment, establishing a multilevel infrastructure for future cancer research	Obesogenic environments including population density, commuting patterns, food outlets, amenities, walkability, and traffic density	Adults aged 45–75 completed a questionnaire for self reported data	Race/ethnicity, SES/income, car usage commute	SES was related to obesity. Lower density of businesses was related to Black and White women, while lower traffic density among White men was also related to obesity.
Anderson 2014, Sydney, Australia [84]	To examine differences between shade covering in playgrounds of higher and lower-socioeconomic-status areas within metropolitan Sydney, Australia	Shade structures (n = 1033) in 139 urban playgrounds	Audit of playgrounds and shade structure no population was examined	SES/income	Lower-SES areas of the city had lower access to shade. Activity areas in playgrounds in the lowest-SES areas had 34% lower mean shade coverage than the highest SES regions.
Duncan 2014, Boston, USA [85]	To examine racial/ethnic and socioeconomic disparities in the tobacco retail environment across neighborhoods in Boston	Tobacco retail and availability	Ecological analysis	Race/ethnicity, poverty	Predominantly Hispanic neighborhoods had higher exposure to tobacco outlets
Marsh 2020, New Zealand [86]	To examine the potential impact of tobacco being available only from pharmacies only, from liquor stores, or only from petrol stations in New Zealand	Tobacco retail and availability	Census aged 15 and older	SES/income	Density of tobacco outlets was higher in low-SES areas.
Tucker-Seeley 2016, Rhode Island, USA [87]	To investigate the association between neighborhood sociodemographic characteristics and tobacco retail outlet density in the state of Rhode Island	Tobacco retail and availability	Ecological analysis	Race/ethnicity, SES/income, education	Tobacco density is negatively associated with income, and education; tobacco density increases with proportion of Black, Hispanic, and poverty.
Kong 2021, USA [24]	To explore whether the racial, ethnic, and socioeconomic composition of a census tract may relate to tobacco retail density	Tobacco retail density	Census data	Race/ethnicity, SES/income, poverty	Higher exposure to tobacco outlets for low-SES and predominantly Black or Hispanic neighborhoods.

## Data Availability

Data sharing not applicable.

## Abbreviations

EPA	Environmental Protection Agency
GEE	Generalized estimating equations
HAP	Hazardous air pollution
NAAQS	National Ambient Air Quality Standards
NATA	National Air Toxics Assessment
NPRI	National Pollutant Release Inventory
OLS	Ordinary Least Squares
SES	Socioeconomic status
TRI	Toxics Release Inventory
UVR	Ultraviolet radiation
